# Indoor radiocaesium contamination in residential houses within evacuation areas after the Fukushima nuclear accident

**DOI:** 10.1038/srep26412

**Published:** 2016-05-23

**Authors:** Hiroko Yoshida-Ohuchi, Takashi Kanagami, Yasushi Satoh, Masahiro Hosoda, Yutaka Naitoh, Mizuki Kameyama

**Affiliations:** 1 Graduate School of Pharmaceutical Sciences, Tohoku University, 6-3 Aramaki-Aoba, Aoba-ku, Sendai, Miyagi 980-8578, Japan; 2 Advanced Industrial Science and Technology, 1-1-1 Umezono, Tsukuba, Ibaragi 305-8568, Japan; 3 Department of Radiological Life Sciences, Hirosaki University Graduate School of Health Sciences, 66-01 Hon-cho, Hirosaki, Aomori 036-8564, Japan; 4 Japan Environment Research Co., Ltd., 02-15-1 Hon-cho, Aoba-ku, Sendai, Miyagi 980-0014, Japan

## Abstract

Indoor contaminants were investigated from July 2013 to January 2015 within ninety-five residential houses in five evacuation zones, Iitate village, Odaka district, and the towns of Futaba, Okuma, and Tomioka. A dry smear test was applied to the surface of materials and structures in rooms and in the roof-space of houses. We found that ^134^Cs and ^137^Cs were the dominant radionuclides in indoor surface contamination, and there was a distance dependence from the Fukushima Daiichi nuclear power plant (FDNPP). For surface contamination in Iitate village (29–49 km from the FDNPP), 24.8% of samples exceeded the detection limit, which is quite a low value, while in Okuma (<3.0 km from the FDNPP), 99.7% of samples exceeded the detection limit and surface contamination levels exceeded 20 Bq/cm^2^ (the value was corrected to March 2011). In residential houses in Okuma, Futaba, and Tomioka, closer to the FDNPP than those in Odaka district and Iitate village, surface contamination was inversely proportional to the square of the distance between a house and the FDNPP. In the houses closest to the FDNPP, the contribution of surface contamination to the ambient dose equivalent rate was evaluated to be approximately 0.3 μSv/h.

The Great East Japan Earthquake (magnitude 9.0) and the subsequent tsunami on March 11, 2011, resulted in major damage to the Fukushima Daiichi nuclear power plant (FDNPP). From March 12 onward, various incidents at multiple units occurred, including explosions which appeared to have been caused by hydrogen at Units 1 and 3 on March 12 and 14, respectively, an explosion incident and smoke at Unit 2, and an explosion and a fire at Unit 4 on March 15^1^,^2^. Major releases of radionuclides occurred in the period from the afternoon of March 12 to the morning of March 16. On the evening of March 12, due to a plume effect, a reading of 20 μSv/h was observed from a measurement made at the joint government building of the City of Minami Soma. It is believed that the plume was initially blown south by a weak northerly wind and then diffused to the north by a strong southerly wind^1^,^2^,^3^. Following an explosion and a fire at Unit 4 on March 15, the plume was very likely to have blown to the south because the wind often blew from the north during this period, and the Japan Atomic Energy Agency in Tokai village^4^,^5^, Ibaraki observed a rise in the dose rate and detected radioactive iodine in the atmosphere.

In the absence of precipitation, the dispersed pollution caused dry deposition during the period in which the radioactive plume passed over the area. Dry deposition occurred not only outdoors, but also indoors. We previously reported the contamination of internal surfaces with an autoradiography image^6^ and assumed it had been caused by wind dispersal when the radioactive plumes moved over this district. Unlike the contamination associated with the Chernobyl accident, we considered that the indoor contamination was not associated with soil particles that had inadvertently been brought in from the garden because the Japanese have a custom of taking off their shoes indoors. The relationship between indoor and outdoor contaminant concentrations depends on the ventilation and indoor deposition rate coefficients^7^. Most Japanese houses are built of wood and are leaky, with poor air-tightness. In these leaky dwellings with a high ventilation rate, the indoor/outdoor concentration ratio would be high^7^. The filtering effect of the building envelop (the penetration of particles into a house) also affects infiltration of airborne pollution into houses. The filtering effect for particles in the size range 0.1 to1-μm is small (less than 10%)^8^, indicating the contribution to indoor contamination due to this effect might be small after the Fukushima accident since the particle size of the Cs carried by sulfate aerosol particles was measured to be approximately 0.5 μm in size^9^. The presence of airborne contaminants in dwellings will lead to deposition on the interior surfaces^7^,^8^. The indoor air concentration is given by the difference between ingression and losses due to exfiltration, cleaning, radioactive decay, resuspension from indoor surfaces, and indoor deposition^8^. Particle resuspension by household residents performing normal activities, such as cleaning, are an important factor in indoor particle concentration, although submicron particles are essentially nonsuspendable under circumstances encountered in residences^10^.

By August 8, 2013, the areas to which evacuation orders were issued were rearranged into three areas (Areas 1, 2 and 3), in response to the annual cumulative dose. Areas 1, 2 and 3 were those to which the evacuation orders were ready to be lifted, in which the residents were not permitted to live, and where it was expected that the residents will have difficulty returning for a long time, respectively^11^. The areas that have been designated as evacuation areas by October 1, 2014 are shown in Fig. 1. At a Cabinet meeting on June 12, 2015, the Japanese government proposed that two categories of evacuation orders (Areas 1 and 2) will be lifted by the end of the 2016 fiscal year (i.e., March 31, 2017)^12^. The government has already notified the town of Naraha, that was designated as Area1 and completely evacuated, and the evacuation order was lifted on Sept. 5, 2015^13^. Decontamination in Area 3 has started in Okuma and the change in classification from Area 3 to Areas 1 or 2 is expected in the future.

For residents intending to make a permanent return to their home, information about indoor deposition is important when assessing the consequences of the nuclear accident in the context of risk assessment. Most individuals spend a large portion of their time indoors and are likely to be exposed to indoor contaminants during their daily life, with doses from long-lived contaminants usually being the major long-term hazards. However, very little information regarding indoor deposition exists and no studies have yet been published that provide measurements of indoor deposition in the Fukushima evacuation areas.

In this study, the indoor surface contamination were investigated in 95 residential houses from July 2013 to January 2015, in five evacuation zones where all administrative districts have been designated as evacuation areas. The levels of indoor radiocaesium contamination on the interior surfaces of the dwelling were evaluated and the differences between the areas investigated were examined. The influence of surface contamination on the ambient dose equivalents was also assessed.

## Results

Through the whole investigation period, 2,653 samples were collected in total within 95 residential houses in Iitate village, Odaka district, and the towns of Futaba, Okuma, and Tomioka, Fukushima Prefecture. The numbers of samples collected in rooms, roof-spaces, and from wooden columns in rooms for each area are summarized in Table 1. For all materials in rooms and roof-spaces, the horizontal surface was rubbed using a smear test paper. For wooden columns, the vertical surface was rubbed. Samples were divided into those exceeding the detection limit (0.004 Bq/cm^2^) and those below the detection limit. The relative frequency distribution of surface contamination for samples collected in rooms, roof-spaces, and from wooden columns in rooms is shown in Fig. 2, which covers all 1,662 samples collected in Odaka district, Okuma and Futaba, and Tomioka. Table 1 shows that 67.1% and 65.5% of samples collected in rooms and in roof-spaces, respectively, exceeded the detection limit; however, only 24.2%t of samples taken from wooden columns in rooms exceeded the detection limit. The same tendency was observed in all areas investigated. Figure 2 shows that the relative frequency distribution of surface contamination for samples collected in rooms was similar to that in roof-spaces, while for wooden columns in rooms there tended to be much smaller levels of surface contamination.

Table 1 also shows a very low percentage (24.8%) of samples exceeded the detection limit in Iitate village, while 89.4, 99.7, and 96.4% of samples exceeded the detection limit in Odaka district, Okuma and Futaba, and Tomioka, respectively. As shown in Table 1, the distance to the FDNPP is shorter in the order of Okuma and Futaba, Tomioka, Odaka district, and Iitate village. The percentage of samples below the detection limit became larger as distance from the FDNPP becomes increased. The relative frequency distribution of surface contamination for all samples collected in rooms is shown in Fig. 3 for each area of Iitate village, Odaka district, Okuma and Futaba, and Tomioka. The distance from the FDNPP is indicated in parenthesis. The results in Okuma and Futaba were divided into two groups: (1) houses located farther away than 3.0 km, and (2) houses located within 3.0 km of the FDNPP. It was clear that the relative frequency distribution indicated a larger surface contamination in homes closer to the FDNPP. In Fig. 3, it should be noted that for surface contamination in Iitate village, 24.8% of samples exceeded the detection limit, which is quite a low value.

In Fig. 4, the values of surface contamination for 27 houses in Odaka district are shown as a box plot in order of their distance from the FDNPP. Number in parenthesis in the measurement’s name represents each distance (km) between the house and the FDNPP. The median surface contamination (black closed square), with an interquartile range of Q1–Q3, which are the middle values in the first and the second halves of the rank-ordered data set, for each residential house in Okuma, Futaba, and Tomioka is plotted as a function of distance between each house and the FDNPP in Fig. 5. An equation by power approximation is shown in the figure, indicating that surface contamination is inversely proportional to the square of distance between a house and the FDNPP. The average outdoor and indoor ambient dose equivalents obtained for each house are also shown in Fig. 5 as blue and green squares, respectively with one standard deviation. The measurements obtained from a house in Okuma, where the decontamination work was completed, were excluded from the figure. There was no relationship between the outdoor ambient dose equivalent and distance from the FDNPP, and between the indoor ambient dose equivalent and distance from the FDNPP, either.

## Discussion

The difference in the relative frequency distribution of surface contamination between samples collected in rooms and those collected from wooden columns shown in Fig. 2 can be explained by the difference in the indoor deposition velocities of radiocaesium between floors (horizontal surface) and walls (vertical surface)^7^,^8^. Contamination on surfaces in the indoor environment is caused by airborne contaminants. The total time-integrated deposition of contaminant nuclides on surfaces increases as the deposition velocity to the indoor surface increases^7^. Lange experimentally showed deposition velocities to floors are six times higher than those to walls for 0.5-μm indium particles and deposition velocities varied depending on aerosols of the size^8^. Based on Lange’s measured values, Andersson *et al.* estimated that the total deposited contamination on floors is six times higher than those on walls for ^134^Cs and ^137^Cs^7^ since the Activity Median Aerodynamic Diameter of aerosols of ^134^Cs and ^137^Cs was measured to be the order of 0.7 μm in various European countries after the Chernobyl accident^14^,^15^. The similar size (approximately 0.5 μm) of the Cs carried by sulfate aerosol particles was observed^9^ after the Fukushima accident as well.

Comparing samples collected in rooms with those in roof-spaces, a similar tendency in the relative frequency distribution of surface contamination was observed, as shown in Fig. 2, indicating that the pollution deposited not only in rooms, but also in the roof-spaces. However, the frequency distribution of surface contamination in roof-spaces indicated less surface contamination than in rooms, with approximately 20% of roof-spaces below 0.01 Bq/cm^2^ compared to 7% of rooms. The difference might have been caused by the difference of furnishing between in rooms and in roof-spaces. The deposition velocity is higher in the furnished rooms than in unfurnished rooms^8^. The difference also might indicate that airborne contaminants entered rooms from an outdoor origin and then diffused into the roof-space due to convective air movements^8^.

Figure 3 clearly shows a distance dependence from the FDNPP, with the levels of surface contamination exceeding 20 Bq/cm^2^ for houses in Okuma (<3.0 km). Terada *et al.* showed the accumulated dry deposition of ^137^Cs from 5 JST on March 12 to 0 JST on May 1 based on simulations made by Worldwide version of System for Prediction of Environmental Emergency Dose Information (WSPEEDI-II)^16^, indicating that the areas corresponding to a large amount of dry deposition were mainly located near the FDNPP and dry deposition gradually decreased with distance from the nuclear plant, with a decrease in ground-level concentration due to atmospheric dispersion. General features can be observed in the results in Fig. 3, although the spatial resolution in the results calculated by WSPEEDI-II was not sufficient to observe differences over several kilometers. The reason for the very low indoor deposition in Iitate village was considered to be not only distance from the FDNPP (29–49 km), but also the altitude (400–700 m), with Iitate village located inland and surrounded by mountains. The other areas investigated all had a flat terrain with a coastal location.

In Fig. 4, the results for 27 Odaka houses are summarized, with no relationship apparent between surface contamination and distance from the FDNPP. The inconsistency between surface contamination and distance from the FDNPP might be derived from small differences in distances between these buildings, as well as differences in local turbulence conditions. The indoor surface contamination for the 27 Odaka houses was discussed in a previous paper^17^, in which a difference in the median values was observed among the districts when the results of the surface contamination for houses were compared as groups in different districts. The groups located closer to the ocean had smaller median values. Figure 4 also indicates a large discrepancy for each individual house. The house referred to as Odaka-24 (16.8) is located next to Odaka-25 (16.8) in the same district of Kanaya; however Odaka-24 had a high median value (0.28 Bq/cm^2^) and Odaka-25 had a lower value (0.05 Bq/cm^2^). We asked the residents how often they return home and whether they clean the room since the accident. Based on their answers, we considered the reason for the discrepancy in surface contamination among houses. Factors such as whether running water is available, how often the residents return home, and the frequency with which residents enter the house may affect the level of surface contamination^17^ as Odaka residents became allowed to visit their houses temporary after Odaka district was rearranged from restricted area to Area 1 and 2 on April 2012.

Figure 5 shows that surface contamination was inversely proportional to the square of the distance between a house and the FDNPP. The three houses closest to the FDNPP (<3.0 km) were located 1.6 km southwest (SW), 1.9 km west-northwest (WNW), and 2.6 km south (S), and had exceedingly high surface contamination. Especially, the two houses located at 1.6 km SW and 2.6 km S (the first and the third one from the left in Fig. 5) had higher surface contamination than the house located 1.9 km WNW of the plan (the second one). A plume with a high-concentration of radionuclides passed over these houses and the surrounding land, causing dry deposition before the concentrations decreased due to atmospheric dispersion. If the aerosols in the areas nearest to the FDNPP had been very large, giving a high gravitational settling over short distances, they might have contributed to surface contamination. Residents of these three houses returned home very rarely as they are located in Area 3 and radiation dose rate have been high, and the indoor environment remains largely as it was just after the accident. The differences in indoor contamination levels may therefore be explained by wind direction, release duration, and the release time of the radioactive plume. Katata *et al.* showed temporal changes in wind speed and direction, and air dose rates observed by monitoring cars around the monitoring posts (MP), gates, and the gym on/near the border of the site of the FDNPP^3^ based on published data from the Tokyo Electric Power Company^18^. It indicated that increases in the air dose rate at the west gate (W) and MP5 (WNW) were observed twice for a short period with relatively low dose rates, while the increases at the main gate (WSW), MP7 (SW), and MP8 (S) provided continuously high dose rates. These temporal changes were consistent with the pattern of surface contamination in the three houses closest to the FDNPP.

Figure 5 also shows the discrepancy between indoor surface contamination (black squares) and the outdoor ambient dose equivalent (blue squares) and between indoor surface contamination (black squares) and the indoor ambient dose equivalent (green squares) as well. The indoor surface contamination was caused by dry deposition and the outdoor dose by wet deposition, which predominantly raised the air dose rate in the yard and the surrounding ground. The indoor dose was almost proportional to the outdoor dose with the ratio of the indoor dose to the outdoor dose (the reduction factor) of 0.4. The areas of wet deposition were distributed heterogeneously in locations far from FDNPP, as well as around the FDNPP^19^, while dry deposition decreased with distance from the plant. It should be noted that the fourth house from the left in Fig. 5, which is located 3.6 km northwest of the FDNPP in Futaba, had a relatively low outdoor ambient dose equivalent, although the indoor surface contamination was relatively high, indicating the ratio of dry deposition to total deposition was relatively high. The WSPEEDI-II simulation results indicated that the ratio of dry deposition to total deposition was relatively high not only near the FDNPP, but also in the northeastern coastal area of Miyagi Prefecture and in the north of Ibaraki Prefecture, although the ratio of wet deposition to total deposition was high in most areas far from the FDNPP^16^.

Exposure to particulate contaminants inside residences can constitute a potential health hazard. Indoor deposits can make a dose contribution to individuals residing in affected dwellings. The possible influence of indoor contamination on the indoor ambient dose equivalents and the reduction factor has been reported^20^,^21^. The results in Figs 3 and 5 indicate that indoor contamination varied substantially depending on the area, with contamination being quite high in houses near the FDNPP. The contribution of surface contamination to the indoor ambient dose equivalent was evaluated for the house 1.6 km SW from the FDNPP. The area source like the contaminated surface can be modeled as a disk source. If the activity is uniformly spread over the area such that gamma-rays are emitted isotropically as Q_s_ (photons/sec/cm^2^), the photon flux, *φ* (photons/sec/cm^2^) at a point h m distant from the center of a disk source with a radius of R m was obtained using the following equation^22^:


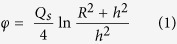


Assuming that radioactivity of 1.0 Bq/cm^2^, emitting n photons per disintegration, is homogeneously distributed in the disk source, the following equation was developed from equation (1) to obtain the ambient dose equivalent rate, H (μSv/h).


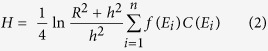


where E_i_ is energy of the gamma-ray (Mev), f(*E*_i_) is the photon emission rate of each gamma-ray, and C(*E*_i_) is the ambient dose equivalent rate per unit flux.

The 1 cm dose equivalent rate constant, Γ (μSv · m^2^/MBq/h), which is the constant providing the 1 cm dose equivalent rate at 1 m distance from the 1 MBq source, was defined as the following equation^23^;


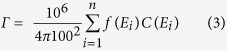


By combining equation (2) with equation (3), the following relationship was obtained.


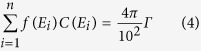


By applying equation (4) to equation (2), equation (5) was obtained.


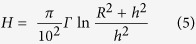


The value of Γ is 0.249 and 0.0927 (μSv · m^2^/MBq/h) for ^134^Cs and ^137^Cs, respectively^24^.

The floor area of the house 1.6 km SW from the FDNPP is approximately 100 m^2^. We assumed that two horizontal surfaces (the room floor and the roof-space floor at a height of 2 m above the floor) of a house with dimensions 10 × 10 × 3 m (h) were homogeneously contaminated, with the median radioactivity observed in the house (8.0 Bq/cm^2^). The room floor and the roof-space floor can be approximated as two disk sources with each radius, R of 5.64 m, which gives the same area of 100 m^2^ as the floor area of the house. Using equation (5), the ambient dose equivalent rate, *H* at a height of 1 m above the room floor for ^134^Cs and ^137^Cs on March 2011 was calculated to be approximately 0.3 μSv/h (R = 5.64 m and h = 1 m, *H* = 1/2 ((*π*/100) × 0.249 × ln ((5.64^2^ + 1^2^)/1^2^) + (*π*/100) × 0.0927 × ln ((5.64^2^ + 1^2^)/1^2^)) × 2 × 8.0)). For this calculation, the effect of the wall was not taken into account.

The average indoor ambient dose equivalent for this house was 9.0 ± 2.2 μSv/h, with one standard deviation (measured values were corrected to March 2011), indicating the contribution of surface contamination to the indoor ambient dose equivalent was 3.0%. This seems quite low, suggesting that the indoor ambient dose equivalent was strongly affected by the outdoor contamination, which was caused by wet deposition. This area had a large rainfall from around midnight to early morning on Mar 16, 2011 (from Analysis of Precipitation, Japan Meteorological Agency) when the radioactive plume flowed from the FDNPP^3^,^18^. However, the contribution of indoor contamination is expected to become larger after decontamination, because decontamination will be conducted outdoors and not indoors. In the same manner, the contribution of radiocaesium surface contamination to the indoor ambient dose equivalents for houses in Tomioka, Odaka, and Iitate was evaluated to be approximately 0.02, 0.01, and 0.004 μSv/h, respectively, assuming that the surfaces are homogeneously contaminated with the highest median radioactivity for each area (0.63, 0.28, and 0.1 Bq/cm^2^, respectively). These values were also relatively low and decreased over time as these were calculated based on surface contamination corrected to March 2011.

Beta particles emitted from ^134^Cs and ^137^Cs have a short range in practically any material. Andersson *et al.* reported that the dose contribution of beta rays to toddlers from the floor could in some cases be an order of magnitude higher than that dose contribution to adults^7^, although the beta dose from nearly all perceivable sources would be negligible at a distance of about one meter from the source. The dose contribution of beta rays should be considered depending on the average height of the toddlers in the house and the dimensions of the room.

The loose and removable surface contamination would cause not only external exposure, but also internal exposure from the intake of radioisotopes by ingestion and/or inhalation. Resuspended contaminants may present a risk for people living in a contaminated area, both through inhalation and deposition on the body. Resuspended particles had a completely different size distribution from that of the initially deposited contaminants^7^. Dose coefficients for the intake of radionuclides vary depending on the Activity Median Aerodynamic Diameter^25^,^26^. Therefore, it is important to characterize the contaminants with respect to their particle size for the evaluation of internal exposure. In addition, it has been experimentally observed that the contamination becomes more strongly attached to a surface over time^7^. This might vary depending on the surface materials used to make floors and furniture. There is no published data indicating the size distribution of aerosols in the samples collected indoors for wooden houses in Fukushima evacuation areas, no data regarding changes in the size distributions of aerosols after resuspension, and no information regarding changes in the levels of attached surface contamination over time, either. Moreover, decontamination of surface contamination is another important issue for residents when they plan temporary access or return home. In the previous paper^17^, we mentioned that surface contamination for wooden materials was removed easily. This result was consistent with the high removal function of 0.75, which was determined in this study. However, decontamination efficiency might vary depending on the surface materials, such as a tatami mat, a carpet and so on. Further measurements and investigations are needed.

## Methods

### Locations of measurements

From July 2013 to January 2015, indoor contamination was measured in 95 residential houses in Iitate village, Odaka district in Minami-Soma, and the towns of Futaba, Okuma, and Tomioka, Fukushima Prefecture, where all administrative districts have been designated as evacuation areas. Futaba and Okuma are close to the FDNPP, adjoining each other, with the FDNPP located over the boundary between them. Odaka district and Tomioka are within a 10–20 km radius north and a 5–15 km radius south of the FDNPP, respectively. Residents of these areas near the FDNPP were evacuated immediately after the evacuation instruction was issued on March 11 and 12, 2011^1^,^2^. In April 2011, areas within a 20 km radius from the FDNPP, including Futaba, Okuma, Tomioka, and Odaka districts were legally defined as restricted area^1^,^2^. The residents were prohibited any access to their homes until the restricted areas were rearranged into Areas 1,2 and 3 responding to the annual cumulative dose^1^,^2^. Iitate village is located relatively far from the FDNPP (29–49 km northwest). The Iitate villagers had about a one month grace period after the village was regarded as the deliberate evacuation area in April 11^1^,^2^.

**Figure 1:**
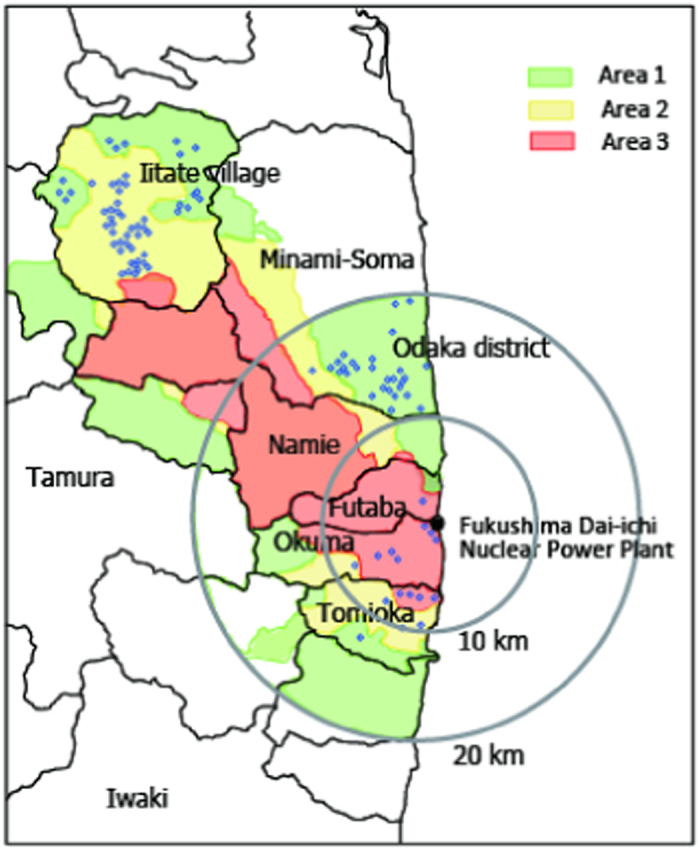
Map of the measurement locations and evacuation areas (October 1, 2014). The locations of the measurements are shown as blue circles. One blue circle indicates one house investigated at each location. The map was created using Microsoft Power Point software (version 14.4.5).

**Figure 2:**
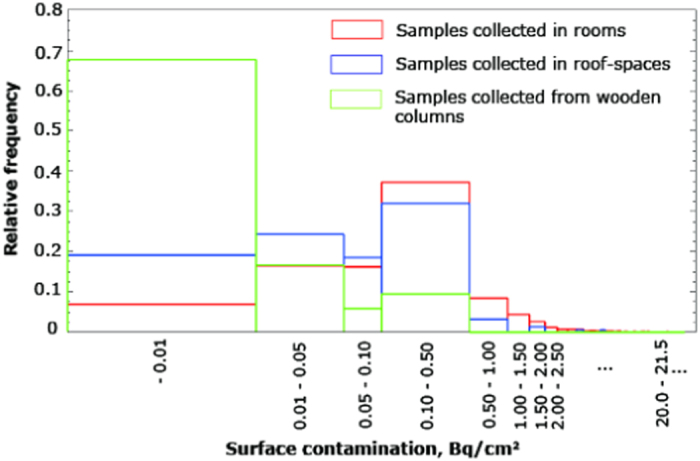
Relative frequency distribution of surface contamination for samples collected in rooms, roof-spaces, and from wooden columns in rooms. It covers all 1,662 samples collected in Odaka district, Okuma and Futaba, and Tomioka.

**Figure 3:**
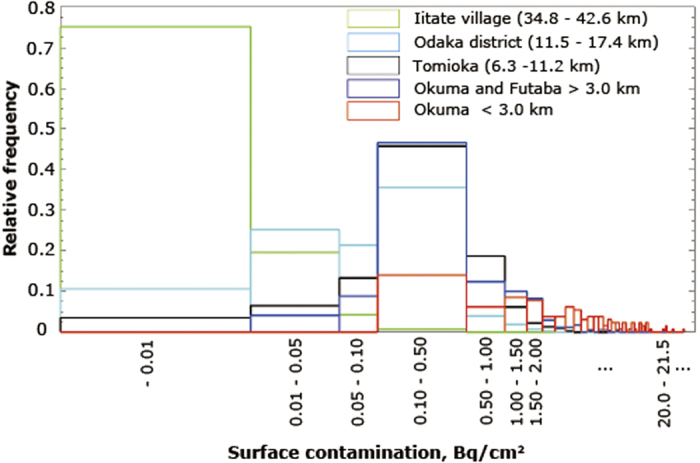
Relative frequency distribution of surface contamination for all samples collected in rooms for each area of Iitate village, Odaka district, Okuma and Futaba, and Tomioka. The distance from the FDNPP is indicated in parenthesis.

**Figure 4:**
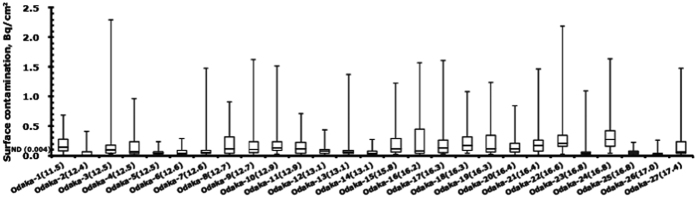
Values of surface contamination for 27 houses in Odaka district, shown as a box plot in order of their distance from the FDNPP. Number in parenthesis in the measurement’s name represents each distance (km) between the house and the FDNPP.

**Figure 5:**
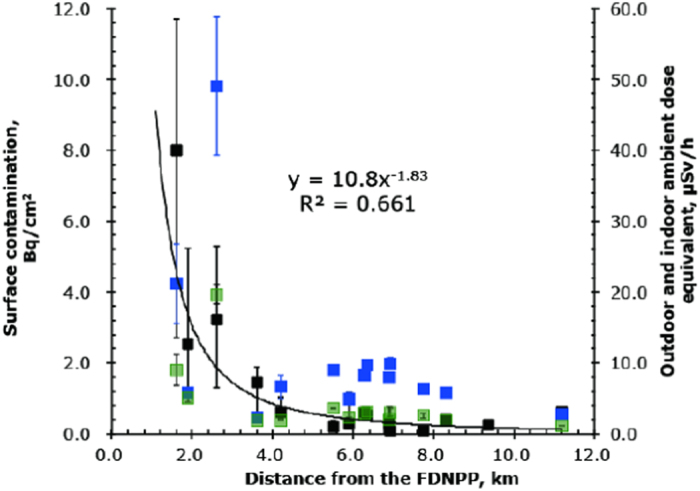
Median surface contamination (black closed square), with an interquartile range of Q1–Q3 for each residential house in Okuma, Futaba, and Tomioka, plotted as a function of distance between each house and the FDNPP. The average outdoor and indoor ambient dose equivalents obtained for each house are also shown in the figure as blue and green squares, respectively with one standard deviation. The measurements obtained from a house in Okuma, where the decontamination work was completed, were excluded from this figure.

A total of 52 houses in Iitate village (16 in Area 1 and 36 in Area 2), 27 in Odaka (26 in Area 1 and one in Area 2), one in Futaba (one in Area 3), seven in Okuma (one in Area 2 and six in Area 3), and eight in Tomioka (two in Area 1, two in Area 2, and four in Area 3) were investigated. The locations of the measurements are shown in Fig. 1 as blue circles. One blue circle indicates one house investigated at each location. Eleven houses were investigated after the decontamination work was completed. Decontamination did not target indoor contaminants, but was conducted for the yard, roof, and gutter. All houses investigated were one- and/or two-story structures and made of wood, except one in Okuma that had a lightweight steel frame.

### Gamma-ray spectrometry of the smear samples

**Figure 6:**
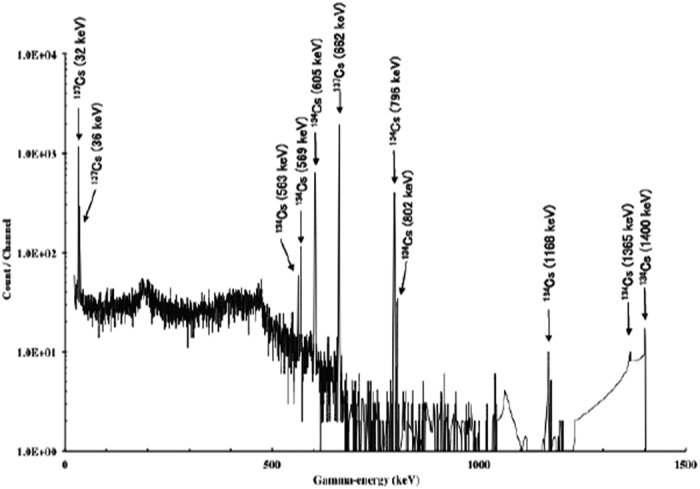
Typical gamma-ray spectrum taken from the samples in Okuma.

Gamma-ray spectra of the smear samples were measured in the laboratory using a high-purity germanium (HPGe) detector ORTEC-GMX-20195-S (AMETEK Inc., Berwyn, USA). A typical gamma-ray spectrum taken from the samples in Okuma is shown in Fig. 6, with gamma-ray emissions from radiocaesium seen at energies of 605 and 796 keV (^134^Cs) and 662 keV (^137^Cs). This figure shows that ^134^Cs and ^137^Cs were the dominant radionuclides in indoor surface contamination after the short-lived radionuclides had decayed, with all sampling and measurements conducted two years after the accident.

The radioactivities of ^134^Cs and ^137^Cs for a part of the smear samples were determined using the same HPGe detector. The full-energy peak efficiency is 2.023% and 2.845% for each peak energy of 796 keV (^134^Cs) and 662 keV (^137^Cs) (see Supplementary Methods).

### Measurements of indoor contaminants

To measure levels of indoor contaminants, a dry smear test was applied to the surface of the materials and structures in rooms and roof-spaces because survey meters cannot provide an adequate estimate of surface contamination under the high radiation background in evacuation areas. An area of 100 cm^2^ of the surface of materials such as wood, metal, glass, and plastic and wood structures were rubbed with moderate pressure using a round smear test paper with a diameter of 2.5 cm. The smear samples were carefully collected from flat, smooth, and non-porus surfaces that had not been cleaned or wiped by residents in every room, with 2,653 samples collected in total. In Supplementary Fig. S1, a typical example of a room and a wooden column investigated is shown with smear sampling positions. For all materials in rooms and roof-spaces, the horizontal surfaces were rubbed. For wooden columns, the vertical surfaces were rubbed. The number of houses investigated and the number of samples collected in rooms, roof-spaces, and from wooden columns in rooms for each area are summarized in Table 1.

The radioactivity on smear test papers was measured for five minutes with a plastic scintillator detector JDC-5300 (Hitachi Aloka Medical, Ltd., Japan) first and then measured with a liquid scintillation counter LS-6500 (Beckman Coulter, Inc., USA) for samples with low counts. The activity was measured using beta rays emitted from ^134^Cs and ^137^Cs, which were the dominant nuclides in the investigated period. We used the plastic and liquid scintillator detector since their detection efficiencies for beta rays emitted from ^134^Cs and ^137^Cs are quite higher than those of the HPGe detector for gamma-rays from them (see Supplementary Methods).

To estimate the total removable surface contamination for beta-emitters, A_sr_ (Bq/cm^2^), the following equation can be used^27^,^28^,


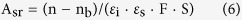


where n is the gross count rate (sec^−1^), n_b_ is the background count rate (sec^−1^), ε_i_ is the instrument efficiency, ε_s_ is the source efficiency, i.e., the fraction of decays within a sample that result in a particle of radiation leaving the surface of the source, F is the removal fraction, and S is the surface area covered by the smear, e.g., 100 cm^2^. The product of the instrument and source efficiency, ε_i_·ε_s_ is the counting efficiency^28^. It was determined by simultaneously measuring a part of the samples using the Ge detector (see Supplementary Methods). Gamma-ray emissions at energies of 796 keV (^134^Cs) and 662 keV (^137^Cs) were measured for 1,000 or 3,000 s. The relationships between radioactivities (^134^Cs + ^137^Cs) measured with the Ge detector and net counts measured with the plastic scintillator detector JDC-5300 and those with the liquid scintillation counter LS-6500 are shown in Supplementary Fig. S2(a,b), respectively. Good linear relationships were observed, resulting in a slope = 0.17, R^2^ = 0.99 and a slope = 0.68, R^2^ = 0.98 for the data in Supplementary Fig. S2(a,b), respectively when a linear fit was established for the data.

The removal fraction, F, might be affected by differences in the nature such as the surface and contaminant particle size distribution. So we determined the removal fraction empirically by the use of repetitive wipes^27^,^28^ in real conditions within the houses in evacuation areas. The repetitive wipes were conducted for 67 surfaces within 12 houses in Odaka district, and the towns of Futaba, Okuma, and Tomioka. An area of 100 cm^2^ of the surface of materials of wood, plastic, glass, and metal (34, 14, 12, and 7 surfaces, respectively) were rubbed three times changing a smear test paper. The ratio, R of n times the removed value of radioactivity to the n-1 times removed value was expressed by the following two equations:


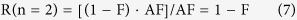



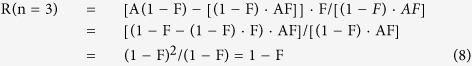


where A is total removal radioactivity.

The relationship between repetitions of the smear and the average ratio of the removed value of radioactivity to the first removed value (n = 1) is shown in Supplementary Fig. S3 with one standard deviation and the equation y = x^−2^ was fitted to the data. The value of F, 0.75 ± 0.16 was determined by this relationship and used as F in this study.

By using the values of the slopes in Supplementary Fig. S2(a,b) and the removal fraction, the total removable surface contamination, A_sr_ was obtained based on the measured values of the plastic and the liquid scintillator detector. All values of radioactivity were corrected to those on March 2011 on the basis of the fact that the ^134^Cs/^137^Cs activity ratio value, which originated from Fukushima is close to 1^29^.

The detection limit, N_d_ is defined as the analyte count that is required to produce a signal greater than three times the standard deviation of the noise level^30^. It was calculated by the following equation,


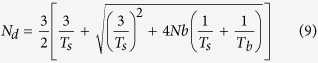


where N_b_ is the background count rate (min^−1^), T_s_ and T_b_ are the counting times of the sample and the background (min), respectively, i.e., 5 min.

The lower detection limit for surface contamination was obtained when the smear samples were measured using the liquid scintillation counter. The detection limit was evaluated to be 0.004 Bq/cm^2^ from equations (6) and (9).

### Measurements of the outdoor and indoor ambient dose equivalent

The ambient dose equivalents [*H* * (10)] were measured outdoors and indoors using a 1” φ × 1” NaI (Tl) scintillation surveymeter TCS-172B (Hitachi Aloka Medical, Ltd., Japan). Outdoor *H* * (10) was measured outdoors at two to four points for each house and at a height of 1 m above the ground. The indoor measurements were collected from two to four rooms where the residents spend much of their time, such as a living room, a bedroom, and a child’s room. Indoor *H* * (10) was measured at the center of the room away from doors and windows at a height of 1 m above the floor. At each point, measurements were collected by changing the direction of the probe of the survey meter to the four directions of east, west, north, and south, and each measurement was repeated three times. The measurement was conducted before the decontamination work started and all values were corrected to those on March 2011.

## Additional Information

**How to cite this article**: Yoshida-Ohuchi, H. *et al.* Indoor radiocaesium contamination in residential houses within evacuation areas after the Fukushima nuclear accident. *Sci. Rep.* **6**, 26412; doi: 10.1038/srep26412 (2016).

## Supplementary Material

Supplementary Information

## Figures and Tables

**Table 1:** The number of samples collected in rooms, roof-spaces, and from wooden columns in rooms for each area and the number of samples exceeded the detection limit (0.004 Bq/cm^2^) and below the detection limit for each area.

Area (Distance from the FDNPP)	Numbers of houses investigated	Total numbers of samples	Samples collected in rooms			Samples collected in roof‐spaces			Samples collected from wooden columns in rooms		
			Numbers of samples	<ND	>ND	Numbers of samples	<ND	>ND	Numbers of samples	<ND	>ND
Iitate village (34.8–42.6 km)	52	991	876	659 (75.2%)	217 (24.8%)	79	51 (64.6%)	28 (35.4%)	36	34 (94.4%)	2 (5.6%)
Odaka district (11.5–17.4 km)	27	991	815	86 (10.6%)	729 (89.4%)	124	29 (23.4%)	95 (76.6%)	52	45 (86.5%)	7 (13.5%)
Okuma and Futaba (1.6–6.9 km)	8	333	299	1 (0.3%)	298 (99.7%)	20	1 (5.0%)	19 (95.0%)	14	3 (21.4%)	11 (78.6%)
Tomioka (6.3–11.2 km)	8	338	308	11 (3.6%)	297 (96.4%)	12	0 (0%)	12 (100%)	18	9 (50.0%)	9 (50.0%)
Total	95	2,653	2,298	757 (32.9%)	1,541 (67.1%)	235	81 (34.5%)	154 (65.5%)	120	91 (75.8%)	29 (24.2%)
