# Supplementary material for: Indoor radiocaesium contamination in residential houses within evacuation areas after the Fukushima nuclear accident
Source: Sci Rep. 2016 May 23;6:26412. doi: 10.1038/srep26412 (PMC4876398; doi:10.1038/srep26412)
Supplement: Supplementary Information [file srep26412-s1.pdf]

Indoor radiocaesium contamination in residential houses within evacuation areas after the Fukushima nuclear accident

Hiroko Yoshida-Ohuchi[*a], Takashi Kanagami[a], Yasushi Satoh[b], Masahiro Hosoda[c], Yutaka Naitoh[d], Mizuki Kameyama[d]

[a]Graduate School of Pharmaceutical Sciences, Tohoku University, 6-3 Aramaki-Aoba, Aoba-ku, Sendai, Miyagi 980-8578, Japan

[b]Advanced Industrial Science and Technology, 1-1-1 Umezono, Tsukuba, Ibaragi 305-8568, Japan

[c]Department of Radiological Life Sciences, Hirosaki University Graduate School of Health Sciences, 66-1 Hon-cho, Hirosaki, Aomori 036-8564, Japan

[d]Japan Environment Research Co., Ltd., 2-15-1 Hon-cho, Aoba-ku, Sendai, Miyagi 980-0014, Japan

Supplementary Information

Methods

Gamma-ray spectrometry of the smear samples

The detection efficiency was calibrated using a standard mixture solution of radionuclides (^{109}Cd, ^{57}Co, ^{139}Ce, ^{203}Hg, ^{113}Sn, ^{85}Sr, ^{137}Cs , ^{88}Y, and ^{60}Co), which was purchased from Eckert & Ziegler Isotope Products, CA, USA. The solution was dropped into a smear test paper with a diameter of 2.5 cm, which is the same size as that of the samples. Then the smear test paper was measured with the HPGe detector to obtain the resulting gamma-ray spectrum and the full-energy peak efficiencies of 2.023% and 2.845% for each peak energy of 796 keV (^{134}Cs) and 662 keV (^{137}Cs), respectively were determined.

Measurements of indoor contaminants

The detection efficiencies of the plastic and liquid scintillator detector are 40%[1)] for the ^{36}Cl standard emitting beta rays with maximum beta energy of 709 keV and 95%[2)] for the ^{14}C unquenched standard emitting beta-rays with maximum beta energy of 156 keV, respectively. Maximum beta energy emitted from ^{134}Cs and ^{137}Cs is 658.39 keV and 513.97 keV, respectively, allowing to use the plastic and liquid scintillator detector with near 40% and more than 95% detection efficiency, respectively.

The total removable surface contamination, A_{sr} (Bq/cm^2), was determined with the Ge detector using the following equation[3),

$$A_{sr} = (n' - n'_b)/(\varepsilon_d \cdot f_i \cdot F \cdot S) \qquad \text{(S1)}$$

where n′ is the gross count rate (sec^{-1}), n'_b is the background count rate (sec^{-1}), ε_d is the detection efficiency, f_i is the photon emission rate, F is the removal fraction, and S is 100cm^2. The full-energy peak efficiencies, ε_d are 2.023% and 2.845%, respectively, and the photon emission rates, f_i are 85.73% and 84.99%[4), respectively, for each peak energy of 796 keV (^{134}Cs) and 662 keV (^{137}Cs).

By comparing equation (6) and equation (S1), the counting efficiency, $\varepsilon_i \cdot \varepsilon_s$ can be obtained as follows.

$$\varepsilon_i \cdot \varepsilon_s = (n - n_b)/((n' - n'_b)/(\varepsilon_d \cdot f_i)) \qquad \text{(S2)}$$

In equation (S2), the right side of the equation can be obtained as a slope evaluated from the relationships between radioactivities measured with the Ge detector and net counts measured with the plastic scintillator detector JDC-5300 and those with the liquid scintillation counter LS-6500.

References

[1)] Hitachi Aloka Medical, Specifications for the plastic scintillator detector JDC-5300 (in Japanese) (2012).

[2)] Beckman Coulter, User Guidelines & Standard Operating Procedure for the Beckman Coulter LS6500 Multipurpose Scintillation Counter (1993).

[3)] Ministry of Education, Culture, Sports, Science and Technology, *Radioactivity measuring method series 7: Gamma-ray spectrometry with a germanium semi-conductor detector*, a revised edition (in Japanese) (1992)

Available at: http://www.kankyo-hoshano.go.jp/series/lib/No7.pdf#search='文部科学省+housyanousokutei'

(Accessed: 8th March 2016)

[4)] Laboratoire National Henri Becquerel, *Table of Radionuclides* (2011)

Available at: http://www.nucleide.org/DDEP_WG/DDEPdata.htm

(Accessed: 8th March 2016)

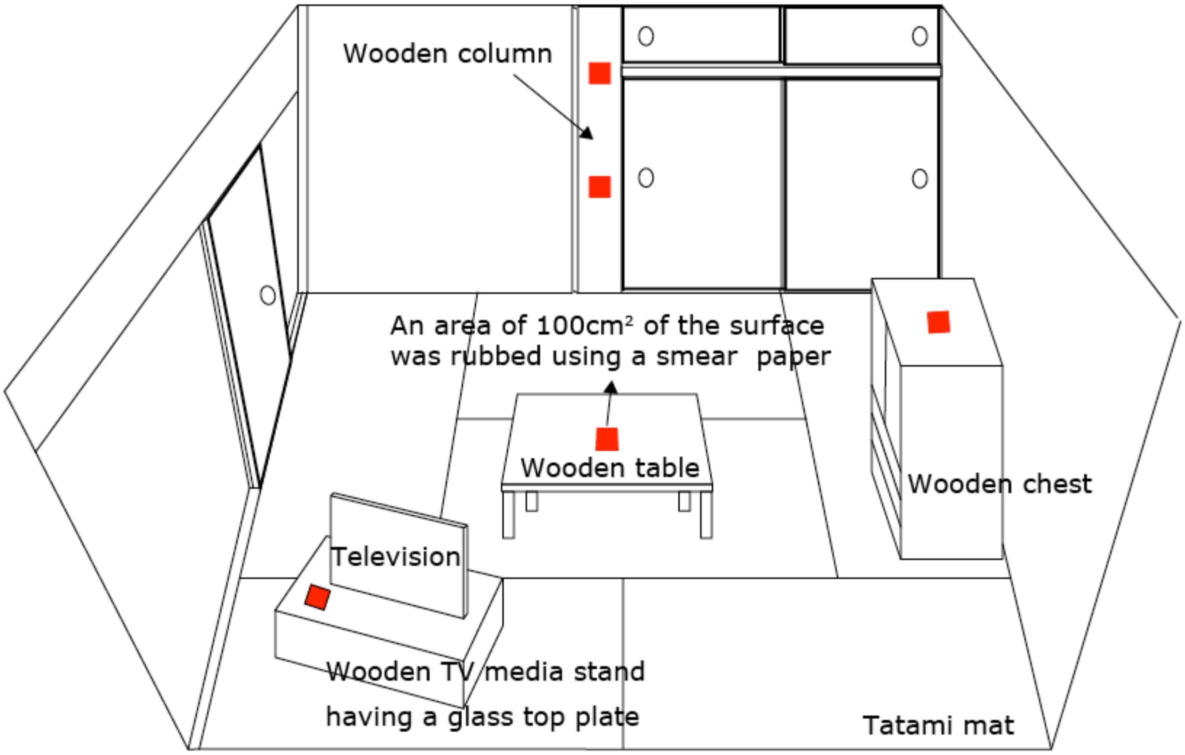

Figs.S1 Typical example of a room and a wooden column investigated with smear sampling positions.

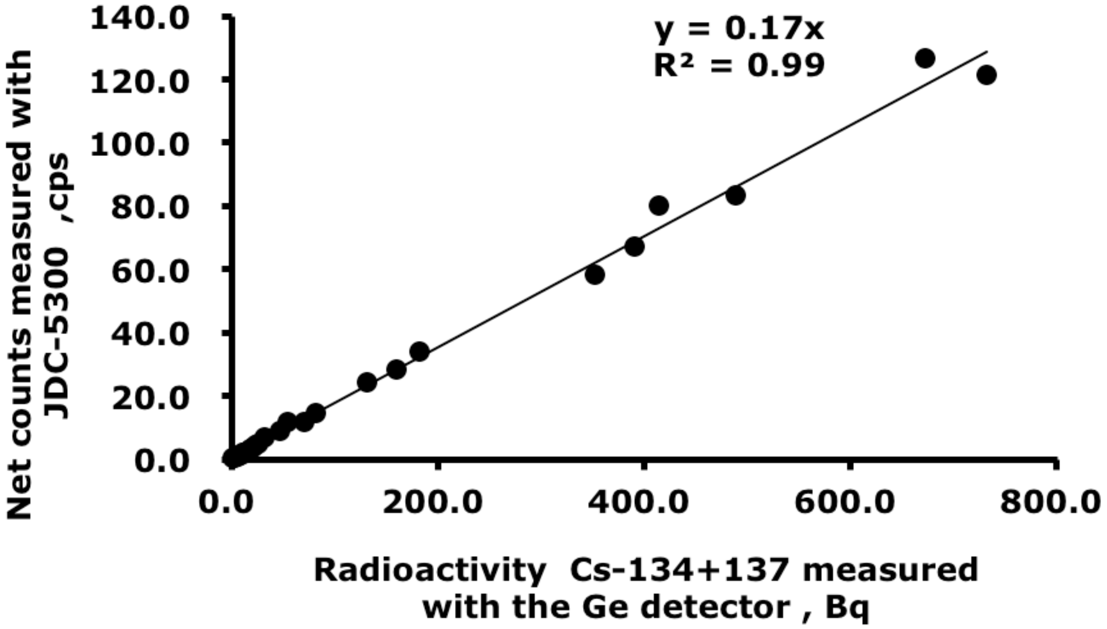

Figs.S2 (a)

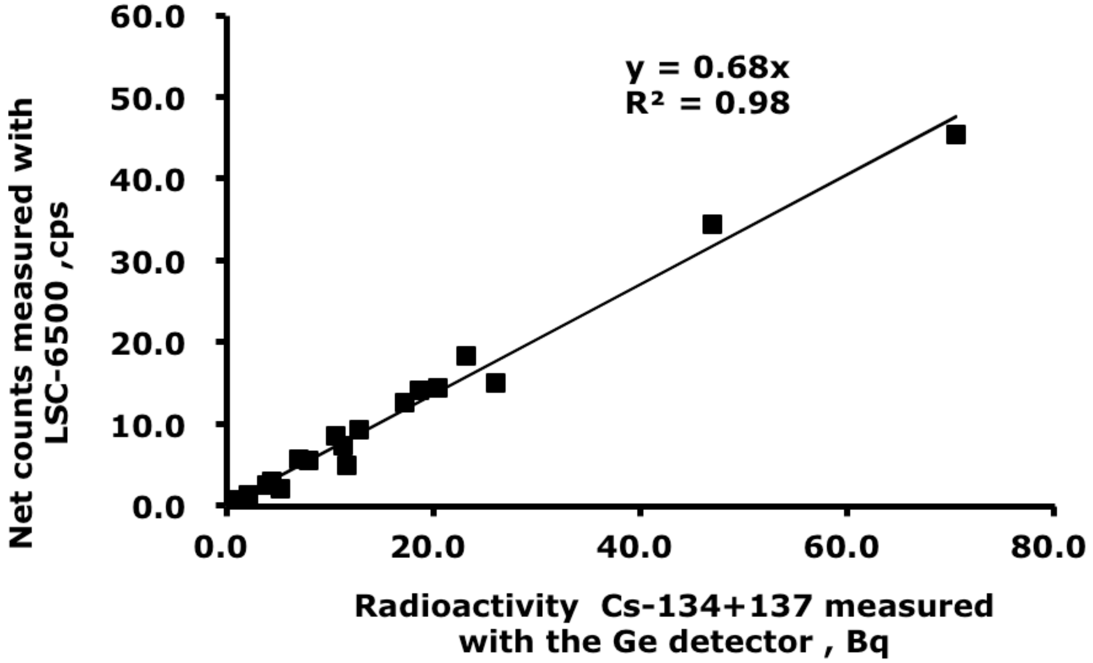

Figs.S2 (b)

Figs.S2 (a) and (b) Relationships between radioactivities (^{134}Cs + ^{137}Cs) measured with the Ge detector and net counts measured with the plastic scintillator detector JDC-5300 (a) and those with the liquid scintillation counter LS-6500 (b).

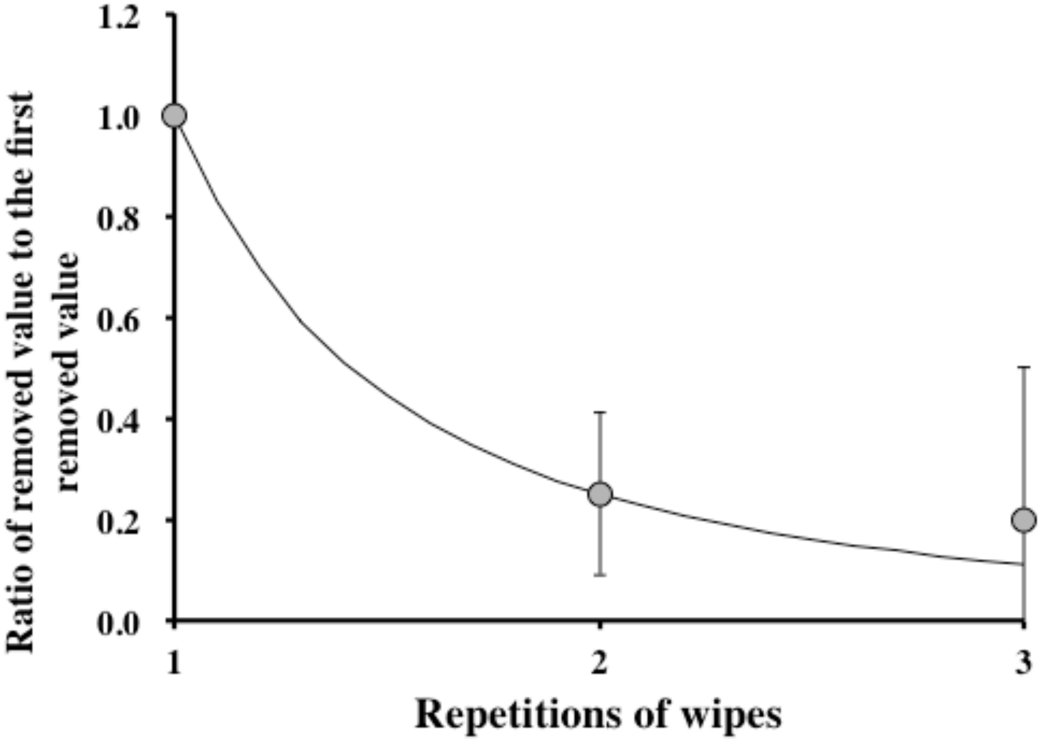

Figs.S3

Fig.S3 Relationship between repetitions of the smear and the average ratio of the removed value of radioactivity to the first removed value.
